# Monitoring of *Chlamydia trachomatis* infection and antibodies in low-prevalence districts of Amhara, Ethiopia: Insights from a hyper-endemic trachoma region

**DOI:** 10.1371/journal.pntd.0013998

**Published:** 2026-02-23

**Authors:** Anna J. Harte, Ambahun Chernet, Getahun Ayenew, Tania A. Gonzalez, Karana Wickens, Sarah Gwyn, Eshetu Sata, Ayalew Shiferaw, Demelash Gessese, Zebene Ayele, Kimberly A. Jensen, Adisu Abebe, Zerihun Tadesse, E. Kelly Callahan, Diana L. Martin, Scott D. Nash

**Affiliations:** 1 Department of Clinical Research, London School of Hygiene and Tropical Medicine, London, United Kingdom; 2 Trachoma Control Program, The Carter Center, Addis Ababa, Ethiopia; 3 Trachoma Control Program, The Carter Center, Atlanta, GeorgiaUnited States of America; 4 Division of Parasitic Diseases and Malaria, Centers for Disease Control and Prevention, Atlanta, United States of America; 5 Health Promotion and Disease Prevention, Amhara Regional Health Bureau, Bahir Dar, Ethiopia; Medical University of Vienna, AUSTRIA

## Abstract

**Background:**

Trachoma, an infectious eye disease caused by *Chlamydia trachomatis* (*Ct*), remains endemic in Ethiopia’s Amhara region despite implementation of the Surgery, Antibiotic, Facial Cleanliness, and Environmental Improvement (SAFE) strategy since 2007. Once a formerly endemic district has reached the trachoma elimination threshold of a trachomatous inflammation-follicular (TF) prevalence of <5% among children ages 1–9 years and mass drug administration has been halted, there is concern that the prevalence could recrudesce back above 5% over time. Formerly endemic districts that are adjacent to still-endemic districts may be at greater risk of recrudescence. This study aimed to evaluate the inclusion of *Ct* infection and antibody testing to complement traditional clinical measures (TF) in Albuko, Bibugn, Debre Birhan Town, and Fagita Lekoma, four low-endemic districts at risk for recrudescence.

**Methodology:**

These four district-level surveys were conducted between March and May 2022. Children ages 1–9 years (n = 2,424) were assessed for TF by certified graders and provided dried blood spots (DBS) for serological analysis. Children ages 1–5 years (n = 1,588) also provided conjunctival swabs to test for *Ct* infection.

**Results:**

The TF prevalence in this age group was < 5% in all four districts. The antibody seroconversion rate (SCR) ranged from 0.1 per 100 children per year in Fagita Lekoma to 1.1 per 100 children per year in Debre Birhan Town, suggesting low transmission compared to proposed low-endemicity thresholds (SCR ≤ 1.6). Further, no positive *Ct* infection cases were detected across the four districts.

**Conclusions:**

The data confirmed that despite being adjacent to trachoma-endemic districts, the four surveyed districts have maintained or achieved the elimination threshold. Additionally, the use of complementary indicators supported clinical evidence of very low disease transmission in these districts. This study highlights that including complementary indicators into surveys provides additional certainty for low-endemicity settings within a region still working towards trachoma elimination.

## Author summary

Trachoma, caused by the bacterium *Chlamydia trachomatis* (*Ct*), is the leading infectious cause of blindness worldwide. Treatment includes mass drug administration of antibiotics, typically delivered at the district level. In Ethiopia, the Amhara region continues to bear one of the world’s highest trachoma burdens, with many districts still showing high levels of active disease. However, some formerly endemic districts that border highly endemic areas may face a risk of trachoma returning after treatment. This study examined whether adding two complementary indicators—*Ct* infection and *Ct*-specific antibodies—alongside traditional clinical indicators could better detail trachoma transmission and assess the risk of recrudescence in four low-endemic Amhara districts. The results demonstrated that these laboratory indicators supported clinical findings of very low transmission, suggesting that these districts have likely achieved elimination targets. By integrating infection and antibody data into routine surveillance, elimination programs can gain a clearer picture of trachoma transmission and make more informed decisions. These findings not only strengthen evidence for sustained elimination in the study districts but also offer a framework for managing other areas nearing the elimination threshold.

## Introduction

Trachoma is an infectious eye disease caused by the bacterium *Chlamydia trachomatis* (*Ct*). Where trachoma is a public health problem, the World Health Organization (WHO) recommends the SAFE strategy: Surgery to correct deviated eyelashes; Antibiotic treatment (distributed through mass drug administration [MDA]); promotion of Facial cleanliness and hygiene; and Environmental improvement [1]. Following WHO recommendations, implementation of the SAFE strategy is based on a prevalence of the clinical sign trachomatous inflammation-follicular (TF) of greater than 5% among children ages 1–9 years in population-based prevalence surveys for mapping at baseline. Using the same methodology, trachoma impact surveys (TIS) are conducted following MDA to assess the prevalence of TF. If a TIS shows TF below 5%, programs stop MDA for a period of two years or more, then conduct a trachoma surveillance survey (TSS) to ensure that the prevalence of TF remains below the threshold, at which point WHO guidelines state trachoma is no longer a public health problem for that indicator [2].

One clearly identified end-game challenge for trachoma programs is the threat of trachoma recrudescence. This has been defined programmatically as a district with at least one TSS at which the TF prevalence among children ages 1–9 years is ≥ 5% [3]. Typically, when this occurs, a district restarts MDA and may receive one or more distributions, along with a follow-up TIS and TSS. Most trachoma endemic countries have experienced recrudescence in at least one district, with the problem particularly acute in Ethiopia, where over half the TSS have returned a TF prevalence above the threshold [4]. Given the repercussions, both in time and money, of restarting MDA programs in recrudescent districts, programs would benefit from having a more complete picture of trachoma transmission in settings close to the elimination threshold.

To better inform programmatic decision-making around the implementation of MDA, it has been recommended that programs consider incorporating the complementary indicators *Ct* infection and *Ct*-specific antibodies [3]. These indicators can provide a better picture of underlying *Ct* transmission and can help provide confidence in TF estimates generated in these settings. These indicators have been collected as part of surveys in a range of endemic settings in Amhara, Ethiopia [5–7]. In 2022, both complementary indicators were incorporated into four surveys within districts close to the elimination threshold in Amhara. The aims of this study were to estimate the prevalence of the clinical signs of trachoma, *Ct* infection, and antibodies to the *Ct* antigen Pgp3 within districts either experiencing or at risk for recrudescent trachoma.

## Methods

### Ethics statement

Ethical approval for this study was granted by Emory University (Emory IRB: 079–2006) and the London School of Hygiene & Tropical Medicine (LSHTM, reference: 16105). The study protocol was also reviewed and approved by Ethiopia’s Ministry of Health, Ministry of Science and Technology, the Amhara Regional Health Bureau, and Tropical Data (https://www.tropicaldata.org/). Prior to conducting surveys, oral informed consent was obtained from village leaders. The study included all individuals ages 1 year and older. Oral consent was received from all participants and parents of children, and oral assent was received from older children. Consent was documented using Android smartphones. Individuals diagnosed with TF and/or trachomatous inflammation-intense (TI) or other suspected bacterial eye conditions received 1% tetracycline eye ointment.

### Setting and historical results

**Fig 1:**
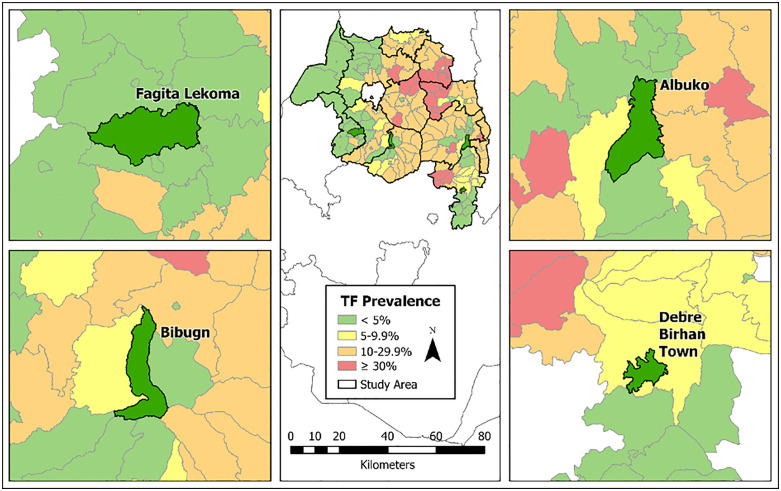
Trachomatous inflammation-follicular (TF) prevalence estimates as of 2022 for the four districts in Amhara, Ethiopia, surveyed in this study, and their neighboring districts. An inset map of the entire Amhara region is included in the center panel. **Map created in ArcGIS Pro 3.3.1 (ESRI, Redlands, CA) using a shapefile sourced from the GADM database of Global Administrative Areas, Ethiopia (ETH).** https://gadm.org**.**

Among surveys conducted between March and May 2022, four districts were purposely chosen for the inclusion of complementary indicators into standard survey procedures, based on a suspicion that they were at risk of recrudescence. All four districts had a demonstrated history of trachoma endemicity, with two districts, Bibugn and Albuko, having had a TF prevalence close to or above 20% at an earlier TIS (S1 Fig) [8–10]. Accordingly, all four have received SAFE interventions and multiple surveys to monitor impact. Three districts, Bibugn, Debre Birhan Town, and Fagita Lekoma, have in general maintained a downwards trajectory of TF prevalence over time since MDA treatments began, had reached a TF prevalence <5%, and thus were due for a TSS in 2022. The fourth district, Albuko, has fluctuated around the 5% threshold over time, and at a 2020 survey met the criteria for recrudescence with a TF prevalence above the 5% threshold at TSS. Per WHO guidelines, one annual round of MDA was administered to Albuko, and the district was due for a TIS in 2022. Because all four districts are adjacent or geographically close to neighboring districts that remain trachoma endemic (Fig 1), it was hypothesized that they may be at enhanced risk for recrudescence.

### Survey design

The number of children required to be enrolled in each district was calculated using the single population proportion formula, described elsewhere [11]. For all surveys, the number of clusters needed from each district was determined by dividing the total targeted number of children ages 1–9 years by the average number of households a team can comfortably enumerate per cluster per day (30), multiplied by the mean number of children ages 1–9 years per household. In Amhara, on average, there are 4.1 persons per household, and approximately 27% of the population is in the 1–9 year age range. Thus, the average number of children ages 1–9 years in a household was estimated at 1.1. Therefore, the number of clusters needed to reach this sample size was 35. However, because WHO does not recommend exceeding 30 clusters per district for this kind of survey, a 30-cluster survey with 30 households per cluster was used for each district [12].

The cross-sectional survey design was conducted at district level, with two-stage probability proportional to estimated population size sampling employed. In the first stage, 30 clusters (gotts) were selected using systematic random sampling. In the second stage, survey teams divided the clusters into segments (locally known as development teams) of approximately 30 households, and one segment was randomly selected to be surveyed. All households in the selected segment were included in the survey.

### Training of graders and recorders

The training of graders and recorders followed the Tropical Data Training Manual [11]. In brief, graders completed a five-day course, passing a slide test (kappa ≥0.7) and a field reliability test (kappa ≥0.7 for TF grade) to qualify. Recorder trainees received intensive training on survey tools, interview techniques, and data recording using Android phones, requiring a score of ≥90% on a standard test to participate in the surveys. Data collectors and technicians involved in sample collection participated in classroom and field practice on consent, collecting samples while maintaining sterile conditions, and maintaining cold chain [6,13]. Graders, recorders, and technicians (tubers) then participated in pilot exercises within communities not involved in the survey.

### Data collection

Each individual age 1 year or older from whom consent/assent was obtained was examined by certified graders for signs of TF, TI, and trichiasis (upper and lower eyelid), following the WHO simplified grading system for trachoma, using 2.5X magnifying binocular loupes, and sunlight or a flashlight for illumination [14]. Where trichiasis was present in an eye, the presence or absence of trachomatous scarring (TS) was also recorded, and the subject or guardian was asked if they had ever been offered management from a health care worker at a primary, secondary, or tertiary health unit for the respective eyelid(s) with trichiasis. Individuals who were affected by trichiasis but had not been identified, diagnosed, or managed by healthcare services were defined as “unknown to the health system”.

A standardized questionnaire was used to collect information required to determine the prevalence of water and sanitation within the study households, and a visual inspection of the household latrine and water, sanitation, and hygiene (WASH) facilities was also conducted. All data were captured electronically using a purpose-built Open Data Kit-based Android smartphone application.

### Blood spot sample collection and analysis

All consenting household members ages 1 year and older had a fingerstick and their blood (60µl) was collected on filter paper. Filter papers were labeled with a bar code, dried, and stored in individual Ziploc bags at -20 °C until testing. Dried blood spots (DBS) were shipped to the United States Centers for Disease Control and Prevention (CDC) at ambient temperature for testing by multiplex bead assay (MBA) as previously described [15]. In brief, DBS were eluted 1:40 in phosphate-buffered saline containing 0.5% casein, 0.3% Tween 20, 0.02% sodium azide, 0.5% polyvinyl alcohol, 0.8% polyvinylpyrrolidone, and 3 µg/mL *E. coli* extract (Buffer B). Eluates were diluted to 1:400 in Buffer B and incubated with Pgp3-coated beads to detect antibody to the *Ct* antigen Pgp3 on a Luminex instrument (Luminex Corp., Austin, TX) equipped with Bio-Plex Manager 6.0 software (Bio-Rad, Hercules, CA). The results from the Luminex were converted to median fluorescence intensity (MFI) with background (Buffer B alone) subtracted out (MFI-BG). Cutoffs for positivity were generated as previously described using a receiver operating characteristic curve analysis of specimens previously classified as positive or negative [5,15,16]. The positive threshold for Pgp3 was 440 MFI-BG.

### Conjunctival swab sample collection and analysis

Conjunctival swabs were collected from children ages 1–5 years as collection among this age group has shown to lead to high response rates, and targets the group considered the main reservoir of infection [17–20]. To collect the swab, the left eyelid was held in the everted position, and the grader wiped the swab across the upper tarsal conjunctiva with a gloved hand. The swab was drawn firmly in one direction over the conjunctiva with enough pressure to cause blanching of the conjunctival vessels, rotated 120 degrees along its axis, and then drawn again firmly across the conjunctiva. The swab was rotated again, and the conjunctiva was swabbed for a third time and placed into dry cryovials. A negative field control (“air swab”) swab was taken on a randomly chosen 5% of monitored children, where a sterile swab passed within one inch of the child’s conjunctiva without making contact. Swab samples taken in the field were stored in a vaccine carrier with ice packs changed regularly. The swabs were transported to the Trachoma Molecular Laboratory at the Amhara Public Health Institute and stored at -20 °C until polymerase chain reaction (PCR) analysis. Within each district, swabs were pooled, five samples per pool, and PCR was performed using an assay that targets two highly conserved regions, using the RealTime PCR assay (Abbott Molecular, Des Plaines, Illinois) on the Abbott m2000 system as previously described [13].

### Data analysis

TF prevalence was estimated, and 95% confidence intervals (CI) were constructed using published methods [21]. Seroprevalence was calculated using the survey design function in R (R Core team, 2024), with results separated by age group and sex. Seroconversion rates (SCR) were estimated using a generalized linear model (GLM) with a complementary log-log link function, which is appropriate for modeling the probability of seropositivity over age. This model assumes a constant force of infection (λ), consistent with an exponential model of seroconversion [22]. The GLM was fitted with an offset based on age, allowing the estimation of the force of infection per year, taking into account the age distribution of the population. The model used has been described previously [5]. SCR is represented by the number of children seroconverting per 100 children per year. Further survey analysis was conducted using the survey package, and robust standard errors for the SCR were computed with the sandwich package [23,24]. Data were mapped using ArcGIS Pro v3.3.1. A small number of serology samples collected from Debre Birhan Town were lost before they could be processed. As a result, the clinical data from the individuals missing serology data were analyzed using the Kruskal-Wallis test and chi-square test to detect whether there were substantial differences between age and sex of those with and without serology data within that district.

## Results

A total of 10,290 participants were examined across the four study districts. A total of 2,653 children ages 1–9 years were examined for clinical signs, and of these, 2,425 individuals provided DBS. Among children ages 1–5 years, 1,588 provided a conjunctival swab. The sex ratio was approximately 50% in the 1–9 years age group across all four districts but was slightly skewed in the age 10 + years group, with around 60% female individuals (Table 1). A total of 379 individuals were missing from the serology dataset, with the majority (94%) from Debre Birhan Town. Of these 379, 86 (22.7%) were children ages 1–9 years. Among the missing data from this district, participant age ranged from 1–84 years, with 61.1% being female. No statistically significant differences were observed between those missing serology samples and those with available samples, for both age (P = 0.78) and sex (P = 0.33).

**Table 1:** Sample sizes and proportion of people examined in four districts of Amhara, Ethiopia, 2022.

District	Total households	Total people enumerated	10 + y examined (% enumerated)	10 + y examined female (% total)	10 + y DBS (% examined)	1–9 y enumerated	1–9 y examined (% enumerated)	1–9 y examined female (% total)	1–9 y DBS (% examined)	1–5 y Eye swab (% 1–9 y examined)
Albuko	900	3046	1975 (79.4)	1128 (57.1)	1860 (94.2)	558	549 (98.4)	263 (47.9)	522 (95.1)	319 (58.1)
Bibugn	899	3053	1832 (77.6)	1124 (61.3)	1641 (89.6)	692	678 (98.0)	338 (49.9)	628 (92.6)	420 (61.9)
Debre Birhan Town	902	2982	1780 (75.6)	1081 (60.7)	1379 (77.5)	629	609 (96.8)	306 (50.2)	495 (81.3)	369 (60.6)
Fagita Lekoma	900	3444	2050 (78.6)	1226 (59.8)	1939 (94.6)	835	817 (97.8)	392 (48.0)	780 (95.5)	480 (58.8)

y=years; DBS=dried blood spots.

Household access to water within 30 minutes varied across all four districts, ranging from 40.6% in Albuko to 80.0% in Fagita Lekoma. Access to any latrine was greater than 60% in all four districts; however, access to an improved latrine was low in general, ranging from 0% in Albuko to 9.6% in Debre Birhan Town (Table 2).

**Table 2:** Prevalence and 95% confidence intervals (in parentheses) of water, sanitation, and hygiene variables in four districts of Amhara, Ethiopia, 2022.

District	Access to water within 30 minutes	Access to an improved water source	Access to a latrine	Access to an improved latrine
Albuko	40.6 (37.0–44.2)	59.2 (55.5–62.8)	72.8 (69.4–75.9)	0 (0–0)
Bibugn	76.3 (73.1–79.3)	33.3 (29.9–36.9)	78.3 (75.0–81.2)	1.6 (0.9–2.8)
Debre Birhan Town	43.2 (39.2–47.4)	74.9 (71.1–78.4)	63.3 (59.2–67.2)	9.6 (7.4–12.5)
Fagita Lekoma	80.7 (77.6–83.4)	30.6 (27.4–34.1)	61.4 (57.6–65.0)	3.8 (2.7–5.3)

The first number represents the prevalence, and the numbers within parentheses represent the 95% CI intervals.

**Fig 2:**
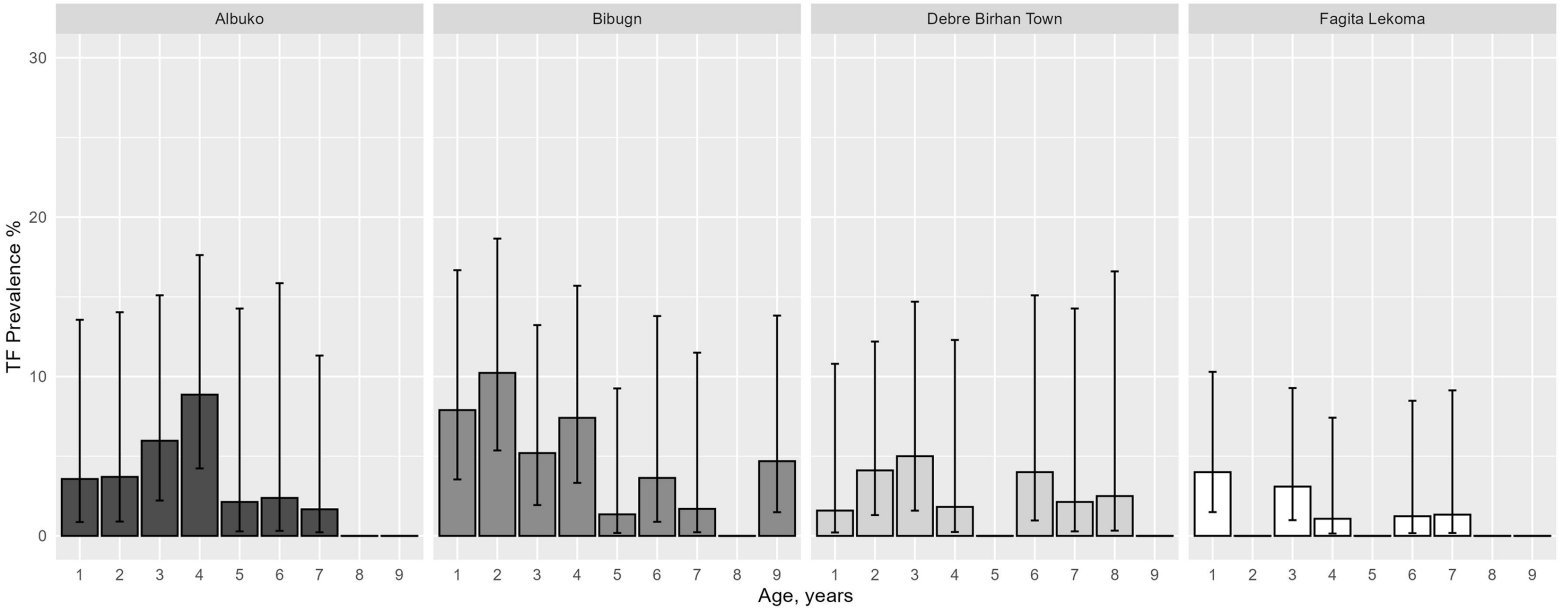
Age-specific prevalence of trachomatous inflammation-follicular (TF) among children ages 1–9 years (n = 2653) in four districts in Amhara, Ethiopia, 2022. Error bars mark 95% Confidence Intervals.

The prevalence of TF among children ages 1–9 years was less than 5% in all four districts (Table 3). TI was less than 1% across all four districts, and trachomatous trichiasis (TT), unknown to the health system, was above the 0.2% TT threshold in all four districts, ranging from 0.45% (95% CI: 0.22–1.1) in Debre Birhan Town to 0.95% (95% CI: 0.51–1.48) in Bibugn. The TF prevalence by age among children is shown in Fig 2. There were zero positive cases of *Ct* infection as determined by PCR in any district. None of the 120 air swabs were positive by PCR.

**Table 3:** District-level prevalence of clinical and serological indicators among children ages 1–9 years in the four selected districts of Amhara, Ethiopia, 2022.

District	Zone	Most recent round of MDA (years since last MDA)	Total rounds of MDA	Survey type	Pgp3 prevalence % (CI)	TF prevalence % (CI)	TI prevalence % (CI)	TT unknown to the health system prevalence % (CI)
Albuko	South Wollo	Mar 2021 (1)	7	TIS	3.1 (1.9–5.0)	2.8 (1.3–5.0)	0.2 (0–0.6)	0.46 (0.23–0.74)
Bibugn	East Gojjam	Jan 2019 (3+)	11	TSS	4.6 (3.2–6.6)	3.7 (1.6–6.0)	0.2 (0–0.4)	0.95 (0.51–1.48)
Debre Birhan Town	North Shewa	May 2018 (3+)	9	TSS	4.9 (3.3–7.1)	1.3 (0.2–2.4)	0.3 (0–0.8)	0.45 (0.14–0.87)
Fagita Lekoma	Awi	Jan 2019 (3+)	11	TSS	0.8 (0.4–1.7)	1.0 (0.4–1.7)	0.2 (0–0.5)	0.91 (0.47–1.44)

MDA = mass drug administration; TIS = trachoma impact survey; TSS = trachoma surveillance survey; CI = confidence interval; TF = trachomatous inflammation-follicular; TI = trachomatous inflammation-intense; TT = trachomatous trichiasis among individuals >= 15 years.

**Fig 3:**
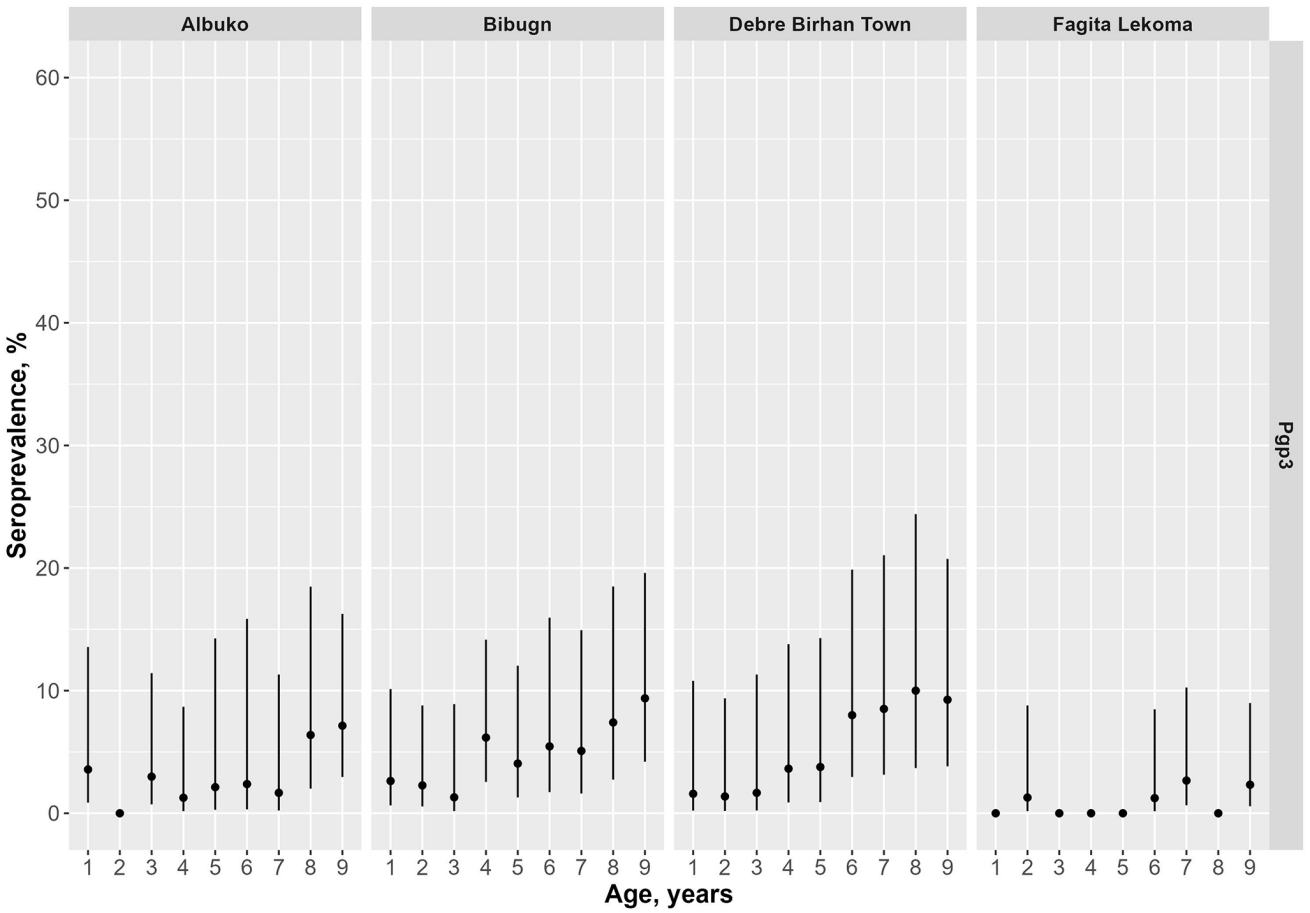
Age-specific seroprevalence of *Chlamydia trachomatis* Pgp3 among children ages 1–9 years (n = 2425) in four districts in Amhara, Ethiopia, 2022. Error bars mark 95% Confidence Intervals.

**Fig 4:**
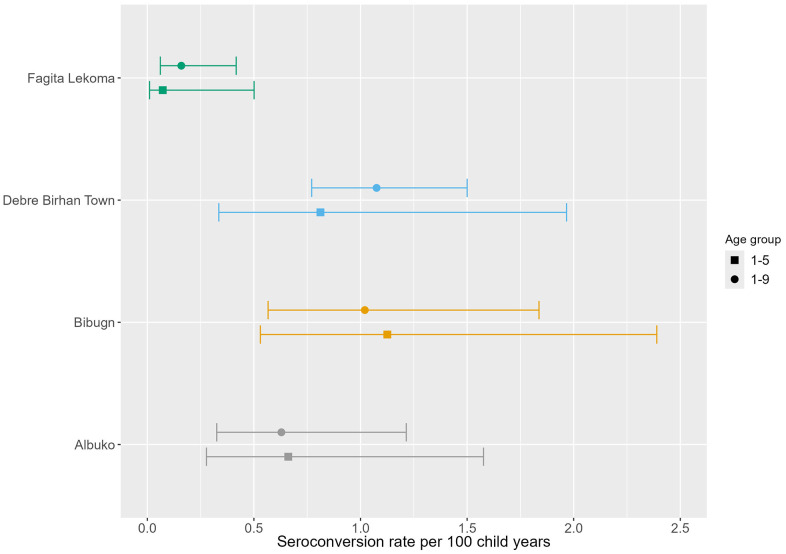
The seroconversion rate per 100 child years for the *Chlamydia trachomatis* Pgp3 among children ages 1–5 years and 1–9 years in Amhara, Ethiopia, 2022. Error bars mark 95% Confidence Intervals.

**Fig 5:**
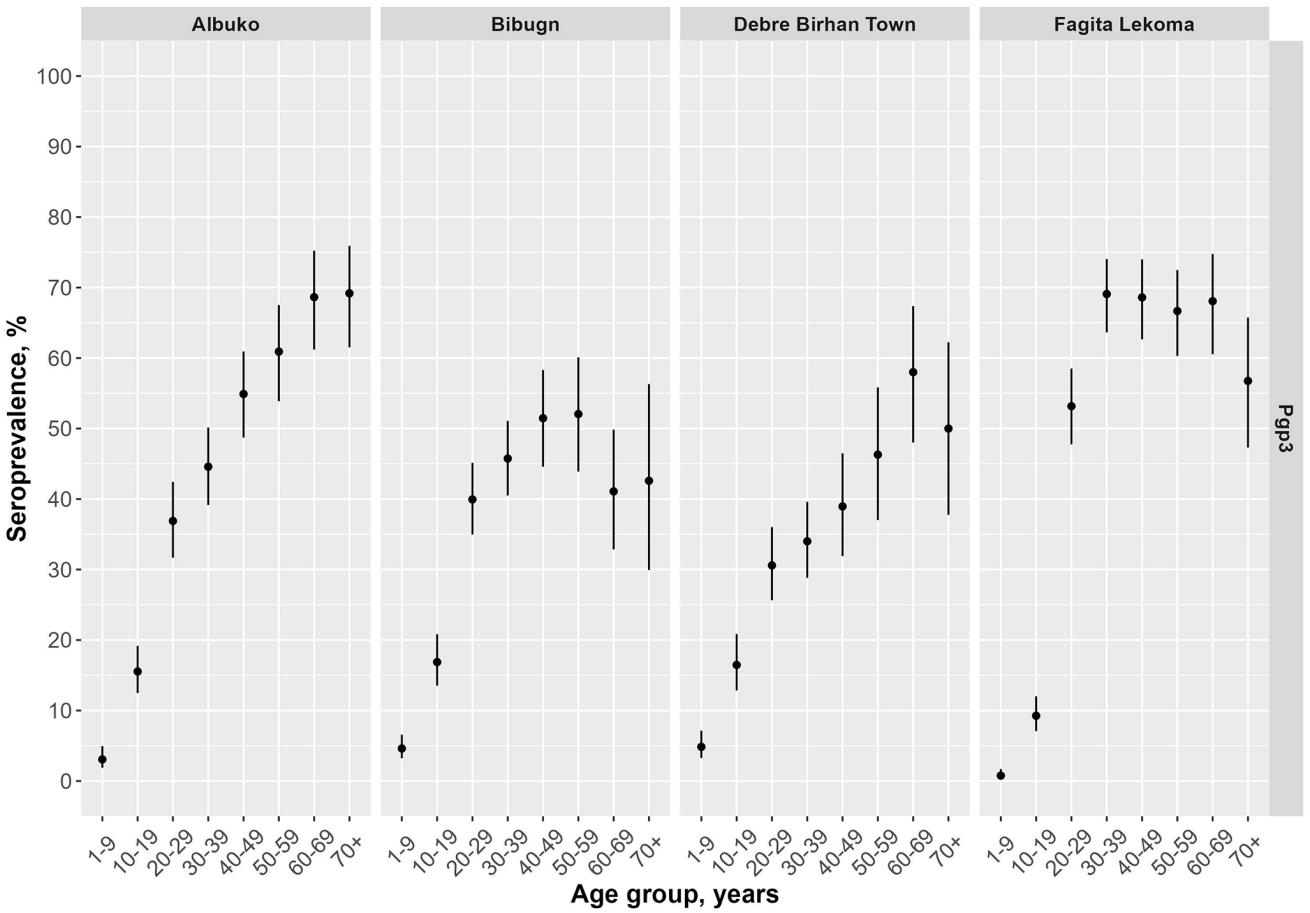
Seroprevalence for *Chlamydia trachomatis* Pgp3 across all ages (n = 9244) in four districts in Amhara, Ethiopia, 2022. Error bars mark 95% Confidence Intervals.

The prevalence of Pgp3 antibodies among children ages 1–9 years ranged from 0.8% (95% CI: 0.4–1.7) in Fagita Lekoma to 4.9% (95% CI: 3.3–7.1) in Debre Birhan Town (Table 3 and Fig 3). The MFI-BG by age is shown in S2 Fig. The SCR among children ages 1–9 years, ranged from 0.2 (95% CI: 0.1–0.4) in Fagita Lekoma to 1.1 (95% CI: 0.8–1.5) in Debre Birhan Town (Fig 4 and S1 Table). For children ages 1–5 years, the SCR ranged from 0.1 (95% CI: 0.0–0.5) in Fagita Lekoma to 1.1 (95% CI: 0.5–2.4) in Bibugn. Seroprevalence among all ages demonstrated a general increase with age, reaching above 50% in Fagita Lekoma by age 20–29 years and in Albuko by 40–49 years (Fig 5).

## Discussion

The four districts chosen for this study were believed to be at risk of trachoma recrudescence due to historically high prevalence, proximity to endemic districts, and previous recrudescence. The results of this study demonstrated that all four districts remained at a TF prevalence below the WHO elimination threshold of 5%, three of which have maintained this low prevalence after approximately three years without MDA interventions. The complementary indicators *Ct* infection and *Ct* antibodies further suggested that trachoma transmission has been interrupted in these four districts, and therefore suggests that recrudescence is unlikely to occur. A recent systematic review using serological data from 45 trachoma endemic or formerly endemic districts estimated that an SCR of ≤1.6 per 100 child years among children ages 1–5 years during a post-baseline survey would be considered as no longer requiring further interventions [25]. These results are encouraging, as Amhara has one of the highest remaining trachoma burdens in the world, with a considerable number of districts with a TF prevalence above 30%. This achievement suggests that in at least some parts of the region, interventions over time had a positive effect in reducing the burden of disease and sustaining reductions.

The Trachoma Control program in Amhara has employed serological monitoring of Pgp3 antibodies since 2017. Initial work conducted by CDC focused on the Chlamydial antigen Pgp3 as it is highly conserved and is detected with high sensitivity and specificity [15,26]. The Pgp3 SCR is a useful indicator for investigating transmission in a population because it provides an objective indicator of recent infections in young children, as well as historical infection in older children and adults. SCR among 1–5 year olds has the potential to most clearly detect recrudescence [22]. In this study, the SCR among children ages 1–5 years and among children ages 1–9 years were similar and were below the 1.6 threshold for all four districts. There is substantial heterogeneity in MDA and TF prevalence history between the four districts, which may have influenced antibody decay rates, however the SCR was well below the proposed threshold regardless. This strongly supports the clinical findings (TF) that trachoma is no longer a public health problem in these districts. The seroprevalence did increase to above 60% among older adults, which is likely due, in part, to a considerable level of historical trachoma transmission. In addition, because the assay cross-reacts with urogenital strains of *Ct*, all-age serology data should be evaluated with caution. While serological evidence suggests transmission has been halted in these districts, interventions to address the TT burden should continue, and a surveillance plan should be established to detect ongoing incident TT cases after the TT threshold has been achieved.

All conjunctival swabs collected from children ages 1–5 years from all 120 study clusters were negative by PCR. The Trachoma Control Program in Amhara has relied on the Abbott Realtime *Ct* assay and the m2000 platform for testing samples since 2014 [13]. The assay is highly sensitive and specific, and the lab remains committed to intense quality control efforts. Previous studies in the region have demonstrated that when TF prevalence is < 10%, *Ct* infection is also very low or not present [6,13]. However, sporadic *Ct* cases have been detected in Amhara even when TF was < 5%, and the epidemiological importance of low levels of *Ct* infection has yet to be determined. The development of programmatic thresholds for the *Ct* infection indicator would be helpful to the global program, and research should continue to develop those thresholds through modeling studies and enhanced understanding of platform-to-platform variability. Simpler PCR tests that require less laboratory infrastructure would also be helpful [27].

Trachoma transmission was low in all four study districts, despite geographical proximity to endemic districts and suboptimal WASH conditions. Three districts were geographically adjacent to districts with a prevalence ≥5% TF (Albuko, Debre Birhan) or >10% TF (Bibugn). This study did not assess the travel patterns between these districts to understand the risk of reintroduction of *Ct* infection to a formerly endemic area, and in general, this type of research is rare [28–30]. Further, while increased WASH services would improve the quality of life within these districts, it is important to note that transmission is now low in these districts despite the observed low household prevalence of improved water and or improved latrines. Interventions for improving WASH remain important in this part of Ethiopia; however, in randomized trial conditions, the improvement of WASH services has yet to demonstrate beneficial effects on trachoma [31]. Although TF prevalence was low overall, 85 cases of TF were still identified through clinical screening. The lag between infection clearance and resolution of the clinical signs of trachoma has been well demonstrated, with the kinetics of infection suggested as the reason for discordance between the two measures, rather than problems with either detection system [32–34]. There is also the possibility that what was recorded as TF was not in fact caused by *Ct* infection, as has been suspected in other parts of the world [35]. Operational research is encouraged to better understand the etiology of the TF response in the absence of *Ct* infection.

The results of this study should be considered in light of several limitations. Approximately 15% of participants in Debre Birhan did not have DBS available for testing. While the results of the sensitivity analysis did not suggest that participants with lost samples were statistically significantly different than those with assayed samples, it is not possible to be sure what effect those missing samples would have had on prevalence and SCR estimates. As noted above, through the use of Pgp3 antibodies, it is not possible to distinguish between ocular and urogenital chlamydia, and therefore, interpretation of the all-age data should be done with caution [15]. Unfortunately, quality data are not yet available on urogenital chlamydia at population levels for these communities to aid in interpreting these data. However, within the 1–5 year old age range targeted for trachoma monitoring, urogenital chlamydia is less likely a factor. Further, without a formal, WHO-recommended SCR threshold for serology or *Ct* infection thresholds, it is difficult to make programmatic decisions that are consistently implemented across countries based solely on these complementary indicators. However, considerable research on this topic is ongoing, and consortia of relevant organizations are in the process of decision-making around operational thresholds [25]. With the very low prevalence of *Ct* infection and Pgp3 SCR observed in these districts, it is likely that they would meet any officially adopted thresholds. This study took advantage of a commercial high-throughput PCR assay which targets two highly conserved loci on the *Ct* plasmid [13]. Despite this, it is possible that PCR assays could miss positives due to target site mutations, although this has been demonstrated to be rare [36,37]. Lastly, the serological testing was conducted by MBA at the CDC in Atlanta, USA. While the MBA requires specialized equipment and expertise, a Pgp3 lateral flow assay (LFA) provides a more feasible option for in-country testing, allowing for faster generation of antibody data and cost savings on shipping samples to the CDC for testing [38].

This study provided actionable data for the programmatic response within these four districts. In the three districts that received TSS, according to WHO guidelines, MDA or further surveys are no longer needed, as active trachoma (TF) is now under control in these areas [2]. The program should continue to work with WASH partners to improve conditions through F and E efforts and should continue TT surgical efforts to reduce the surgical backlog. The three districts make good candidates for case searching using full geographic coverage (FGC) in lieu of another survey, and these efforts should be done under the guidance of the new Ethiopian Ministry’s recommendations for FGC [39]. The survey in Albuko was a TIS conducted approximately 12 months after the last MDA. The results in this district clearly showed low TF and very low transmission, suggesting no further MDA will be needed. Per WHO recommendations, a TSS should be conducted after two or more years to monitor the long-term stability of these results. Albuko had been below the elimination threshold twice before (2010 and 2018), followed by >5% recrudescence at TSS (2014 and 2020). While a TF above 5% at TSS defines recrudescent programmatically, it does not mean *Ct* transmission has returned to a district. Programmatic recrudescence could be due to statistical variability inherent in survey estimation of TF around a threshold [40,41]. In fact, the most recent TF results from Albuko were 3.5%, 5.9% and 2.9% respectively. Therefore, the upcoming TSS should also include complementary indicators, so the program can have a clearer understanding of transmission in this once recrudescent district. Political unrest and insecurity in Amhara, however, have meant that it has not yet been possible to conduct a TSS in this district (as of July 2025). For all four districts, the program should consider innovative long-term surveillance strategies to monitor recrudescent trachoma. While these guidelines have yet to be developed, the incorporation of complementary indicators within existing public health campaigns or services may be one solution.

## Conclusions

Although the Ethiopian Trachoma Control Program has been implementing the SAFE strategy for nearly two decades in some parts of the Amhara region, many of the districts throughout the region remain endemic. This study demonstrated that districts in some parts of the region which have been beneficiaries of SAFE implementation have been successful in reaching the elimination threshold and that these gains were sustainable once MDA has been stopped. Continued interventions and surveillance in TT-endemic districts are recommended to clear the backlog and ensure elimination, even where transmission has stopped. The incorporation of *Ct* infection and antibody data into routine surveys has supported the clinical data, providing more concrete evidence that these districts have reached the elimination threshold for active trachoma. These complementary indicators should be more widely considered to better understand the epidemiology of trachoma near the elimination threshold.

## Supporting information

S1 Fig History of trachomatous inflammation-follicular (TF) prevalence over time for four districts in Amhara, Ethiopia, 2007–2022. The horizontal red line represents the 5% TF threshold. The dotted lines between the baseline and first TIS indicate that baseline surveys were conducted at a zonal level and therefore are not directly comparable to subsequent survey results.
(DOCX)

S2 Fig Median MFI (log MFI-BG) Distribution of Pgp3 by year of age among children ages 1–9 years in Albuko, Bibugn, Debre Birhan Town, and Fagita Lekoma districts, Amhara, Ethiopia, 2022. The horizontal blue line represents the cut-off point for seropositivity. The upper and lower bounds of each box represent the first and third quartiles of the interquartile range, respectively. The horizontal line within each box represents the median. Each black dot represents one individual.
(DOCX)

S1 Table Seroconversion rates (SCR) per 100 child-years and 95% confidence intervals (CI) of Pgp3 among children ages 1–9 years and 1–5 years in four districts in Amhara, Ethiopia, 2022. (DOCX)
