# Supplementary material for: Monitoring of Chlamydia trachomatis infection and antibodies in low-prevalence districts of Amhara, Ethiopia: Insights from a hyper-endemic trachoma region
Source: PLoS Negl Trop Dis. 2026 Feb 23;20(2):e0013998. doi: 10.1371/journal.pntd.0013998 (PMC12952645; doi:10.1371/journal.pntd.0013998)
Supplement: S1 Fig — The horizontal red line represents the 5% TF threshold. The dotted lines between the baseline and first TIS indicate that baseline surveys were conducted at a zonal level and therefore are not directly comparable to subsequent survey results. (DOCX) [file pntd.0013998.s001.docx]

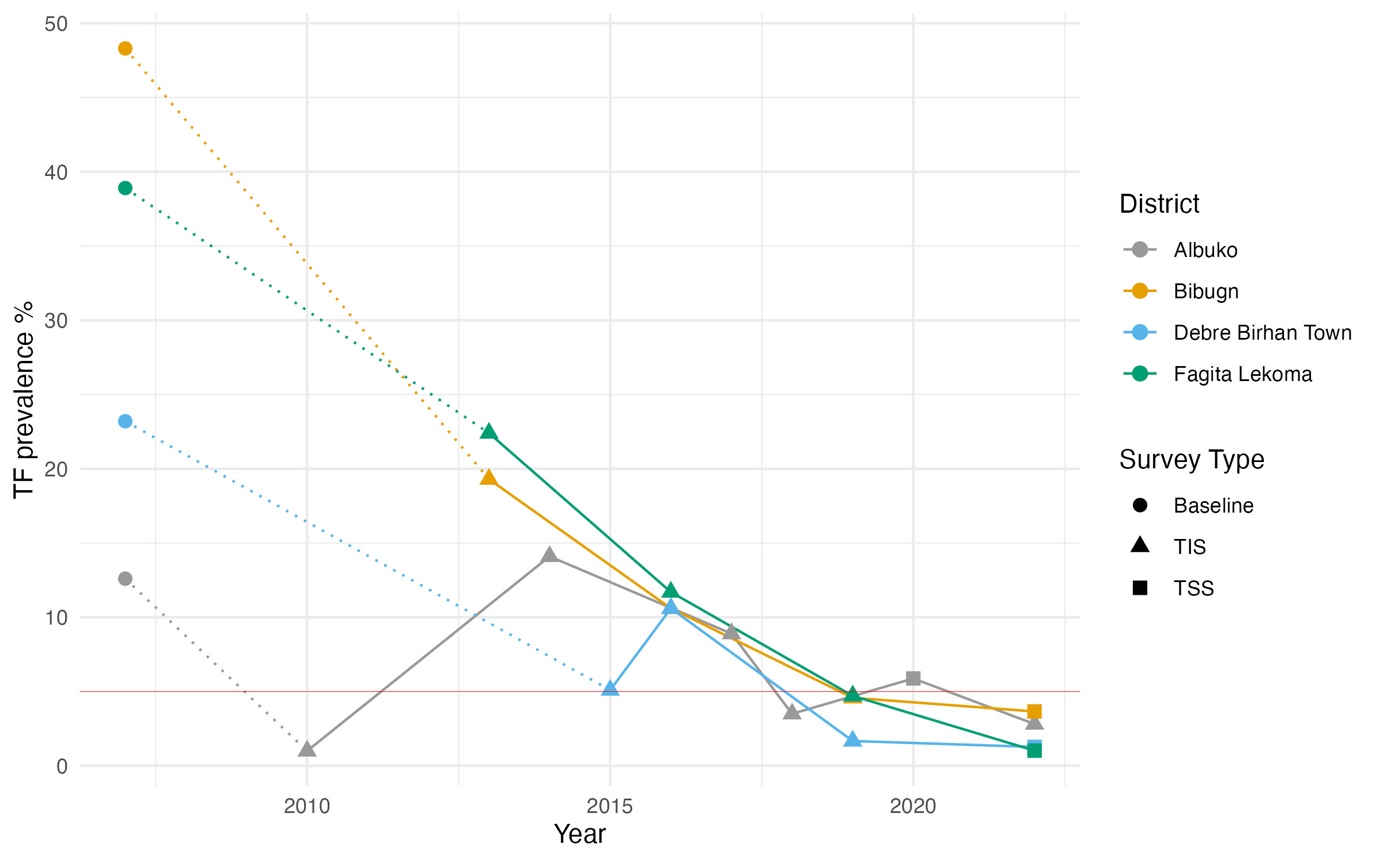


S1 Fig. History of trachomatous inflammation-follicular (TF) prevalence over time for four districts in Amhara, Ethiopia, 2007–2022. The horizontal red line represents the 5% TF threshold. The dotted lines between the baseline and first TIS indicate that baseline surveys were conducted at a zonal level and therefore are not directly comparable to subsequent survey results.
