# Supplementary material for: Monitoring of Chlamydia trachomatis infection and antibodies in low-prevalence districts of Amhara, Ethiopia: Insights from a hyper-endemic trachoma region
Source: PLoS Negl Trop Dis. 2026 Feb 23;20(2):e0013998. doi: 10.1371/journal.pntd.0013998 (PMC12952645; doi:10.1371/journal.pntd.0013998)
Supplement: S2 Fig — The horizontal blue line represents the cut-off point for seropositivity. The upper and lower bounds of each box represent the first and third quartiles of the interquartile range, respectively. The horizontal line within each box represents the median. Each black dot represents one individual. (DOCX) [file pntd.0013998.s002.docx]

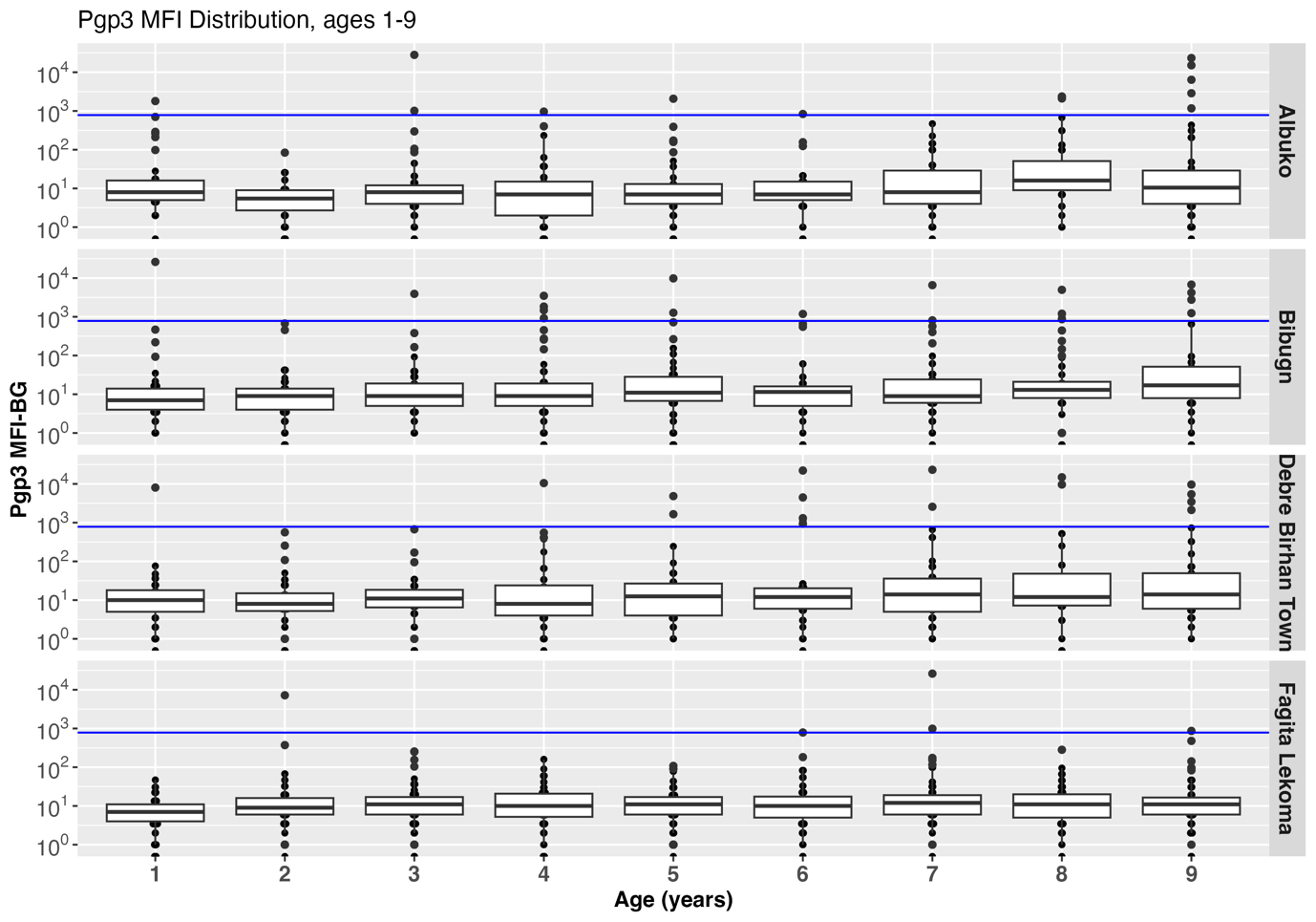


S2 Fig. Median MFI (log MFI-BG) Distribution of Pgp3 by year of age among children ages 1–9 years in Albuko, Bibugn, Debre Birhan Town, and Fagita Lekom districts, Amhara, Ethiopia, 2022. The horizontal blue line represents the cut-off point for seropositivity. The upper and lower bounds of each box represent the first and third quartiles of the interquartile range, respectively. The horizontal line within each box represents the median. Each black dot represents one individual.
