# Supplementary material for: Monitoring of Chlamydia trachomatis infection and antibodies in low-prevalence districts of Amhara, Ethiopia: Insights from a hyper-endemic trachoma region
Source: PLoS Negl Trop Dis. 2026 Feb 23;20(2):e0013998. doi: 10.1371/journal.pntd.0013998 (PMC12952645; doi:10.1371/journal.pntd.0013998)
Supplement: S1 Table — (DOCX) [file pntd.0013998.s003.docx]

S1 Table. Seroconversion rates (SCR) per 100 child-years and 95% confidence intervals (CI) of Pgp3 among children ages 1–9 years and 1–5 years in four districts in Amhara, Ethiopia, 2022.

| **District** | **Pgp3 1**–**9 SCR (CI)** | **Pgp3 1**–**5 SCR (CI)** |
| --- | --- | --- |
| Albuko | 0.6 (0.3–1.2) | 0.7 (0.3–1.5) |
| Bibugn | 1.0 (0.6–1.8) | 1.1 (0.5–2.4) |
| Debre Birhan Town | 1.1 (0.8–1.5) | 0.8 (0.3–1.9) |
| Fagita Lekoma | 0.2 (0.1–0.4) | 0.1 (0.0–0.5) |
